# Zapotin, a Polymethoxyflavone, with Potential Therapeutic Attributes

**DOI:** 10.3390/ijms222413227

**Published:** 2021-12-08

**Authors:** Jakub W. Strawa, Katarzyna Jakimiuk, Michał Tomczyk

**Affiliations:** Department of Pharmacognosy, Faculty of Pharmacy with the Division of Laboratory Medicine, Medical University of Białystok, ul. Mickiewicza 2a, 15-230 Białystok, Poland; jakub.strawa@umb.edu.pl (J.W.S.); katarzyna.jakimiuk@umb.edu.pl (K.J.)

**Keywords:** zapotin, flavonoids, polymethoxyflavone, chemistry, biological activity

## Abstract

The use of plants as traditional medicines is common and has prevailed in many different cultures over time. Polymethoxyflavones (PMFs) are natural polyphenols from the group of flavonoids. Zapotin, a member of the PMFs, is found mainly in citrus plants and is almost exclusively limited to their peels. The chemical structure of zapotin has been questioned from the very beginning, since the structure of flavonoids with a single oxygen atom in the C2′ position is extremely rare in the plant kingdom. To clarify this, the structural determination and bio-inspired synthesis of zapotin are discussed in detail in this review. Due to the broad biological potential of PMFs, the complication in the isolation process and characterization of PMFs, as well as their purification, have been estimated by adapting various chromatographic methods. According to available data from the literature, zapotin may be a promising curative agent with extensive biological activities, especially as a chemopreventive factor. Apart from that, zapotin acts as an antidepressant-like, anticancer, antifungal, and antioxidant agent. Finally, accessible studies about zapotin metabolism (absorption, distribution, metabolism, excretion, and toxicity) underline its potential in use as a therapeutic substance.

## 1. Introduction

Plants are boundless sources of bioactive substances and persist as unfailing tools for discovering new remedies [1]. Polymethoxyflavones (PMFs) are natural polyphenols from the group of flavonoids; they are most commonly found in citrus plants such as orange, bergamots, mandarins, grapefruits, limes or tangerine peels, and their biological activities. Which have been shown to be anti-inflammatory, antioxidant, anti-cancer and anti-atherogenic, have been broadly evaluated in recent years [2,3,4]. PMF is a term for a flavone that bears at least two methoxy groups on its basic benzo-γ-pyrone structure. HPMFs (hydroxypolymethoxyflavones) are PMF derivatives whose chemical structures are determined by the presence of a hydroxyl group instead of a methoxy group at the C5 position. Their occurrence in plant material is almost exclusively limited to citrus peels [4]. Due to the broad biological potential of PMFs and HPMFs, the complications in the process of their isolation and characterization, as well as that of their purification, have been assessed and overcome by adapting various chromatographic methods, e.g., SFC (supercritical fluid chromatography), reversed-phase high-performance liquid chromatography (HPLC), or chiral HPLC [5,6]. It is worth mentioning that the chemical nature of PMFs makes them more lipophilic than hydroxyl flavones, which affects their bioavailability by facilitating the passage of the blood-brain barrier and, thus, the multidirectional pharmacological effects [4]. Additionally, it was observed that the acetylation of PMFs at the C5 position, e.g., 5-acetyloxy-6,7,8,4′-tetramethoxyflavone, results in a more effective antitumor agent than its parent structure [7]. Zapotin, a member of the PMFs with the chemical name 5,6,2′,6′-tetramethoxyflavone, was isolated for the first time from *Casimiroa edulis* [8]. According to available data from the literature, it is suggested that zapotin may be a promising therapeutic agent with broad biological potential, especially as a chemo-preventive factor [9].

Despite the fact that the different bioactivities (e.g., anticonvulsant, antidepressant-like, anticancer, antianxiety, antifungal, antioxidant) of zapotin have been established, there are no distinctly organized review articles available. Thus, this paper summarizes the findings on the techniques used for the analysis, isolation, and separation of zapotin, in addition to describing the interesting biological effects and therapeutic activities of this molecule.

## 2. Methodology

This annotated bibliography focuses on the various approaches to studying zapotin. The search databases for this review were Google Scholar, EBSCO Discovery Service, REAXYS Database, SCOPUS, PubMed, MEDLINE, Web of Science, Wiley Online Library, Science Direct/ELSEVIER, and Taylor and Francis Online [10]. All databases were methodically searched for articles, abstracts, conference papers, and books published from 1911 until 2021. Suitable publications were manually chosen from the following searches: zapotin, methoxyflavone, flavone, polymethoxyflavone, chemistry, biological activity, biological potential, natural occurrence, biosynthesis, bio-inspired synthesis, organic synthesis, chromatography, analysis, isolation, identification, nuclear magnetic resonance, traditional use, pharmacological, natural product, secondary metabolites, therapeutic agent, quantitative analysis, qualitative analysis, sleeping disorders, sleeping-inducing activity, antitumor, anticancer, anticonvulsant, antianxiety, antidepressant, hypotensive, vasorelaxant, antifungal, and antioxidant. The chemical formulas of the compounds were drawn on the online Chem Draw software (http://chemdrawdirect-cdn.perkinelmer.com, accessed on 10 November 2021).

## 3. Natural Occurrence of Zapotin

**Figure 1:**
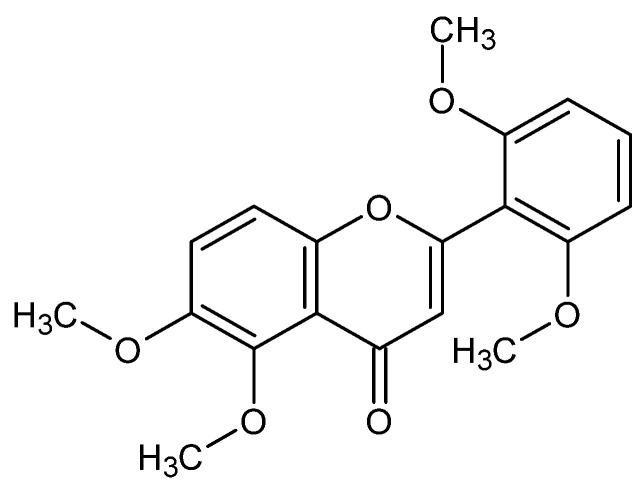
Chemical structure of zapotin.

Reports about the presence of zapotin (Figure 1) in the plant kingdom are limited to only 12 species. Naturally occurring polymethoxyflavones have predominantly been isolated from the *Casimiroa* species, which have been used as a traditional drug to cure various human disorders (Table 1).

In the genus *Casimiroa*, zapotin occurs in *C. edulis*, *C. pubescens*, and *C. greggi*, which was initially classified as *Sargentina greggi* [8,11,12]. *C. edulis* seems to be the best-described species of its genus. Due to its culinary qualities and great ability to adapt to challenging conditions, it is grown in New Zealand, Australia, South Africa, and India’s western regions. This plant is known by the local name “Zapote blanco”, which translates to “white sapote”. Other names are the Mexican apple, Casimiroa, Chapote, Matasano (Spanish), Cacchique (Maya), Ceaxmisttea (Otomi), and Cochitzapoti (Nahuatl) [11]. *C. edulis* fruits are used in gastronomy and serve as an addition to salads and ice creams. Aside from being utilized as food, the leaves and seeds are used in infusions as sedative agents [12,13,14] and painkillers for rheumatism and arthritis [15,16,17]. Furthermore, folk-medicinal reports have mentioned treating diarrhea in children by burning the leaves. On the other hand, the fruit is taken as a remedy for insomnia [14]. The first reports on the chemical composition of *C. edulis* seeds are attributed to José Sanchez and date to 1893. At that time, the presence of alkaloids, resin, essential oil, gums, and sugars was also evaluated [18]. The chemotaxonomic importance of zapotin in *Casimiroa* plants is evidenced by the presence of this compound in large amounts in *C. tetrameria*. This plant is also known as Yuy, and it has been used to treat diarrhea, dysentery, and spastic gastrointestinal conditions [19,20].

The presence of zapotin was also confirmed by GC-MS studies in *Mammea suriga* bark extract. This evergreen tree is endemic and grows in the Karnataka state of India. It is a plant that is valued for its aromatic qualities; in particular, its flowers are used in religious ceremonies and cosmetology, as well as in traditional medicine in Asia. An extract made with the use of petroleum ether in a Soxhlet apparatus allowed researchers to obtain a lipophilic residue, which, in the GC-MS analysis, showed a slight content of zapotin. It is worth mentioning that the GC-MS analysis of *M. suriga* flower buds did not confirm the presence of this methoxyflavonoid [42]. Another South Asian species with zapotin content is *Syzygium alternifolium*. Traditionally, in folk medicine, the shoots, leaves, and fruits of this tree have been used to treat dysentery, joint pain, and gastrointestinal disfunction [49]. In Europe, the identification and isolation of zapotin were carried out by using the leaves of *Primula veris*. Primrose roots display an important role in phytomedicine, the monograph of which can be found in the European Pharmacopoeia [47,50]

## 4. Structural Determination of Zapotin

The chemical structure of zapotin has been questioned from the very beginning. Doubts were raised by the fact that the structure of flavonoids with a single substituent containing an oxygen atom in the C2′ position of the B-ring of the flavonoid is extremely rare in the plant kingdom. Nevertheless, a fusion of demethylzapotin with potassium alkali gave rise to salicylic acid, which strongly suggested that one of the oxygen substituents was at the 2′-position and that the B-ring was unsubstituted in a different manner. It was also considered whether zapotin belongs to the family isoflavones, but this theory was refuted by the high stability of the demethylated flavonoid structure in an alkaline environment. Additionally, free demethylation and regeneration of the molecule were allowed with diazomethane [22,51]. The original assumptions regarding the structure of zapotin were based on the similarity of the values of the IR and UV spectra obtained for 5,6,2′-trimethoxyflavone. The presence of oxygen at the C5 position was confirmed by a positive reaction with iron chloride, giving a dark green color, as well as the absence of a signal in the IR spectrum in the 3µ region, which is typical for hydroxyl substitution. However, moieties at C3 and C8 positions were excluded due to the negative effect in the Shinoda reaction and the lack of an effect in the reaction with *p*-benzoquinone, respectively. It was important for researchers that the following compounds with an increasing degree of methylation of molecules were present in the tested plant material: 5,6-dimethoxyflavone, 5,6,2′-trimethoxyflavone, and 5,6,7,2′-tetramethoxyflavone, which was considered at that time to be zapotin [51]. The high yield of salicylic acid obtained as a reaction product from zapotin incorrectly suggested that its structure was that of 5,6,7,2′-tetramethoxyflavone. Scientists challenged the initial findings on the B-ring substituent configuration. They performed a chemical synthesis to obtain 5,6,7,2′-tetramethoxyflavone and correlated the synthesis product with isolated zapotin. They used 2-methoxybenzoyl chloride to acylate the 2-hydroxy-4,5,6-trimethoxyacetophenone molecule. After structural rearrangement, they firstly obtained 2-hydroxy-2′,4,5,6-tetramethoxydibenzoylmethane and, finally, 5,6,7,2′-tetramethoxyflavone. The hypothesis concerning the original structure of zapotin was refuted by the melting points of the compound isolated from *C. edulis* and the synthetically obtained zapotin. Their melting points were significantly different and were 150–151 and 97 °C, respectively. To confirm this hypothesis, the researchers also made an attempt to synthesize 5,7,8,2′- and 3,5,6,2′-tetramethoxyflavones, but again without obtaining the appropriate melting points (mp) [52]. In parallel, Pai et al. made attempts to obtain synthetic zapotin by using a different scheme. In an alkaline environment, they used a reaction of 2-hydroxy-4,5,6-trimethoxyacetophenone—which is the core of the flavonoid molecule—with *o*-anisaldehyde to obtain polymethoxylated chalcone. Next, by using a gel column and oxidation with selenium dioxide, they obtained a compound with a structure that was intended to be a zapotin. The subsequent scheme assumed the transformation of 5,7,8,2′-tetramethoxyflavone into the expected zapotin by transforming the molecule according to the Wessely–Moser method of performing demethylation in hydroiodic acid, followed by reconstruction of the polyhydroxyflavone into 5,6,7,2′-tetramethoxyflavone. Both synthesis products possessed differences in their physicochemical parameters from those of the isolated zapotin. There was a subsequent argument about verifying the structure of this flavone [53]. Considering the above arguments, Dreyer and Bertelli proposed that the zapotin configuration was that of 5,6,2′,6′-tetramethoxyflavone. Evidence was provided by 100 Mc NMR analyses of the isolated zapotin in the presence of deuterated chloroform and trifluoroacetic acid. Trifluoroacetic acid caused the protonation of the carbonyl group. This resulted in a proton shift in the flavonoid molecule in ring A [23]. The final structure of zapotin was confirmed by a synthesis involving 2-hydroxy-5,6-dimethoxyacetophenone, which was reacted in a hot pyridine medium with a precursor that had methoxy substituents in the expected place—2,6-methoxybenzoyl chloride—which led the researchers to obtain a synthesis intermediate: 2-(2,6-dimethoxybenzoyloxy)-5,6-dimethoxyacetophenone. Then, the addition of sodium hydride transformed the molecule into a diketone, which, after dehydration in ethanol (acidified with sulfuric acid), gave a structure that was fully physiochemically compatible with the natural zapotin molecule [54].

## 5. Bio-Inspired Organic Synthesis of Zapotin

The emerging attempts to determine the profile of the pharmacological activity of zapotin have initiated efforts to obtain it through the synthesis of a high-purity standard substance with physicochemical parameters consistent with the naturally isolated and described compound. Murillo et al. used 2-hydroxy-6-methoxyacetophenone as a substrate, which they subjected to Elbs persulfate oxidation. As a result, they received 2-hydroxy-5,6-dimethoxyacetophenone. Then, the molecule was then coupled to the 2,6-dimethoxybenzaldehyde (B-ring precursor). They achieved a temporary structure of chalcone, which was catalytically oxidized in DMSO into zapotin [55]. On the other hand, Maiti et al. proposed a high-throughput synthesis process allowing them to obtain large amounts of zapotin. Acetophenone dissolved in THF was introduced into the reaction medium created by THF and LiHMDS (lithium hexamethyldisilazide). Afterwards, 2,6-dimethoxybenzoyl chloride was added, and the reaction mixture was poured onto ice, acidified with HCl, and extracted with CHCl_3_. The thoroughly dried organic layer was purified on a silica gel column with hexane-EtOAc elution (1:3 *v*/*v*). The analyte obtained was introduced into a mixture of glacial acetic acid with 0.5% sulfuric acid and heated to 100 °C (in argon). Subsequently, the solvents were removed, the residue was dissolved in water, and it was extracted again with chloroform. The organic layer was chromatographed under the above-mentioned conditions. In this way, 82% efficiency of the synthesis process was achieved, thus avoiding the problematic Baker–Venkataraman transformations, which may have resulted in the formation of synthesis byproducts that would reduce the yield [56].

## 6. Spectroscopic Characterization of Zapotin

Zapotin, white crystalline solid (CHCl_3_), mp 146–147 °C (literature: 147–148 °C). Rf = 0.25 (SiO_2_, EtOAc-hexane 3:1); Rf = 0.91, 0.4 (cellulose, TBA, HOAc); UV λ max nm: 255*sh* (shift), 325; +NaOMe: 255*sh*, 295*sh*, 323; +AlCl_3_: 255*sh*, 325; +NaOAc: 258*sh*, 324; +H_3_BO_3_: 259*sh*, 324; IR (neat) 2939, 2840, 1650, 1592, 1475, 1417, 1357, 1281, 1255, 1111 cm^−1^; ^1^H NMR (300 MHz, CDCl_3_) δ 7.35 (t, J = 8.7 Hz, 1 H), 7.25 (d, J = 9.3 Hz, 1 H), 7.16 (d, J = 9.3 Hz, 1 H), 6.59 (d, J = 8.4 Hz, 2 H), 6.26 (s, 1 H), 3.94 (s, 3 H), 3.88 (s, 3 H), 3.75 (s, 6 H); ^13^C NMR (75 MHz, CDCl_3_) δ 177.9, 158.7, 158.2, 152.2, 149.3, 147.4, 131.8, 119.0, 118.5, 114.9, 113.5, 110.9, 103.6, 61.5, 56.8, 55.7; EIMS (*m*/*z*, relative intensity) 342 (M+, 50), 327 (100), 311 (7), 283 (5), 253 (8), 237 (3), 197 (3), 182 (5), 165 (37), 137 (83), 109 (26), 91 (18), 69 (19), 53 (14); HRMS *m*/*z* calcd for (C_19_H_18_O_6_) 342.1103, found 342.1107. Anal. (C_19_H_18_O_6_) C, H [46,56].

## 7. Techniques for the Analysis of Zapotin in Plant Material

Currently, chromatography seems to be the backbone of separation and analysis science, and it is beginning to be used worldwide in all studies and by all pharmaceutical companies [57]. The separation of zapotin through column chromatography with various types of stationary phases and mobile phases is summarized in Table 2.

The possibilities of qualitative and quantitative determination, high efficiency, sensitivity, and high speed of separation are important advantages of analyses performed with high-performance liquid chromatography (HPLC). It is an effectively developing method with a wide range of uses and has been proven to have a key role in the analysis of extracts and fractions from plants. The use of HPLC in zapotin analyses and the conditions for detection and isolation are given in Table 3. Furthermore, progressions in GC systems and the development of new stationary phases have made it possible to scope out proper means of separation in various applications [62]. The sequent method of the analysis of zapotin in plant material, which includes four species, is gas chromatography (GC) (Table 4).

## 8. Biological Activities of Zapotin

The extraction of bioactive compounds from medicinal plants is an essential step in producing plant-derived drugs [63]. Zapotin and extracts or fractions containing zapotin from different medicinal plants, as well as their medicinal properties, which include antioxidant, antiviral, antibacterial, anticonvulsant, anticancer, antianxiety, antifungal, and antidepressant-like effects, are summarized in Table 5.

### 8.1. Antiviral Activity

To examine the antiviral activity, an extract from *C. edulis* seeds containing zapotin was assayed with respect to HIV-1 RT-associated functions. It was revealed that *C. edulis* inhibited RNA-dependent DNA polymerase (RDDP) and RNase H activities in a concentration-dependent manner with IC_50_ values of 0.27 and 2.0 mg/mL, respectively. Nevirapine has been used as positive control [64].

### 8.2. Anticancer Activity

In several examinations, it was exposed that zapotin is a potent anticancer agent. The studies suggested its role as a chemopreventive and chemotherapeutic agent. *C. edulis* extract inhibited the replication of the K562 erythroleukemia cell line, where it showed dose-dependent cytotoxicity in K562 cells with a CC_50_ value of 3.1 ng/mL [64]. A phytochemical LC-MS analysis of methanolic extracts from *Calliandra portoricensis* root bark disclosed zapotin content in it. The results received with the *C. portoricensis* extract showed a significant increase in the percentage of cells in the S-phase in a dose-dependent manner in prostate LNCaP cells (IC_50_ = 2.4 ± 0.2 µg/mL). Furthermore, in the LNCaP cells was observed a three-fold decrease in the expression of Bcl-2 and a four-fold increase in Bax levels, as well as a 3.5-fold reduction in FIR and a 4.2-fold induction of CCR at 10 µg/mL [36]. The assessment of zapotin activity confirmed its selective cytotoxicity against other human cancer cell lines, including human breast cancer (MCF-7), human glioma (U251N), human pancreatic cancer (PANC-1), and human colon cancer (H-116) [7].

The effects of natural and synthetic zapotin (1 µM, 5 or 10 mg/kg BW) were also examined in the SW480, SW620, and HT-29 colon cancer cell lines and in the generation of an aberrant crypt in female CF-1 mice (ACF). A mediated inhibition of growth was shown in the HT-29, SW480, and SW480 cell lines with an IC_50_ of 2.74 × 10^−7^, 2.29 × 10^−7^, and 5.27 × 10^−7^ M, respectively. It was proven that in an assay of aberrant crypt foci, zapotin treatment resulted in a reduction in the number of large ACFs by 87% (5.0 mg/kg BW) and 67% (10.0 mg/kg BW) in CF-1 mice [55].

In another study, zapotin was tested as a potent inhibitor of the induction of ornithine decarboxylase (ODC) activity by 12-O-tetradecanoylphorbol-13-acetate (TPA) by using a T24 cell line with an IC_50_ of 3.4 ± 1.7 μM. This study also demonstrated the inhibition of TPA-induced NF-κB activity in the HepG2 cell line transfected with NF-κB-luciferase plasmid (IC_50_ = 7.6 ± 3.3 μM) and the induction of 50% of the differentiation of the cells at 0.2 μg/mL (ED_50_ 0.5 μM) in HL-60 cells. Using flow cytometry, it was revealed that zapotin up-regulated CD11b, CD13, and CD14 and down-regulated CD15 myeloid markers in HL-60 cells [56].

The potential antitumor properties of zapotin were also tested on the HeLaPKCεA/E subline in MTT, Western blot, and PKC activity tests. In a dose-dependent manner (from 7.5 to 30 mM), zapotin caused an inhibition of the formation of autophagosomes and a drop in microtubule-associated protein 1 light chain 3 protein levels. The gene expression level of a major negative regulator of autophagy was increased, while the expression of the pivotal autophagy genes was decreased [9]. Anti-initiation and anti-promotion protocols were used for a 15-week examination of zapotin activity in a two-stage mouse skin carcinogenesis model. The compound significantly inhibited 7,12-dimethylbenz(a)anthracene/12-*O*-tetradecanoylphorbol-13-acetate-induced mouse skin tumorigenesis with 1, 5, and 10 μmol/mouse concentrations [67].

### 8.3. Antimutagenic Activity

Unfortunately, zapotin possesses weak inhibitory activity in the mouse mammary organ culture (MMOC) system (IC_50_ = 50 µg/mL). It is worth noting that the IC_50_ of DMBA-induced mutagenesis with *S. typhimurium* strain TM677 was over 40 µg/mL, and the inhibition of ethoxyresorufin *O*-deethylase (EROD) activity with microsomes from the liver of Aroclor-1254-pretreated rats was not determined [58].

### 8.4. Vasorelaxant Activity

To study the vasorelaxant and antihypertensive activity, Froldi et al. determined the arterial dilatation induced by extracts obtained from various species of *Casimiroa*, and the zapotin content in each extract was determined by using an HPLC system. It was indicated that extracts from *C. edulis*, *C. pubescens*, and *C. calderoniae* were generally the most potent samples. After administration of the extracts from *C. edulis*, *C. calderoniae*, and *C. pubescens* (20 µg/mL), the dilatation of arterial tissues reached 86.1 ± 2.5%, 95.4 ± 0.9%, and 82.2 ± 6.6%, respectively. The vascular mechanisms of action depended on the M_3_ muscarinic receptor subtypes with the activation of cGMP-dependent NO signaling [34].

### 8.5. Antimicrobial Activity

Zapotin, which is present in pomegranate peel (PMP) extract in traces (0.46%), was also studied as an antifungal and antibacterial agent. El-Seideek et al. exanimated the diameters of the inhibition zones of *S. aureus*, *S. typhi*, *E. coli*, *A. flavus*, *A. parasiticus*, *A. niger,* and *P. digitatum*. Nevertheless, the extract did not show sufficient activity [44].

### 8.6. Antidepressant-like Activity

Using the forced swim test (FST), researchers investigated the antidepressant-like activity of hexane (HCP), ethyl acetate (ECP), and methanol (MCP) extracts of the roots of *Casimiroa pubescens* in a mouse model. Doses of HCP at 60 mg/kg, ECP at 120 mg/kg, and MCP at 90 mg/kg induced a significant reduction in the FST immobility time. In this study, the first dose was administered 60 min before testing, and the second dose was administered 24, 7, and 1 h before testing. It is worth mentioning that the triple administration of the extracts provided a stronger effect than the single administration [60].

## 9. ADMET of Zapotin

To fully understand the efficacy and safety of zapotin administration, several in vitro and in vivo studies were used, and the dispositions of various metabolites were determined with HPLC-MS. The hepatic metabolism of the investigated compound was studied by using human liver microsomes and human hepatocytes, and was then analyzed with liquid chromatography–mass spectrometry (LC-MS) or liquid chromatography–tandem mass spectrometry (LC-MS-MS). An evaluation of the metabolic stability in human liver microsomes exposed a half-life of zapotin at t_0.5_ = 6 min. It was proved that zapotin undergoes an extensive biotransformation in phases I (seven metabolites) and II (five metabolites) due to hydroxylation, *O*-demethylation, and conjugation. The measure of the zapotin level in rat serum, liver, mammary gland and perirenal fat, and to qualitatively detection of phase I and phase II metabolites led to the conclusion that alteration of zapotin was observed in serum and tissue samples, counting sulfates which were not detected in incubations with human hepatocytes. Furthermore, after administration of zapotin (in a dose of 40 mg/kg BW/day) in rats for three days, no clinical signs of toxicity were observed [69].

## 10. Conclusions

The emerging research on the medicinal properties of zapotin in terms of its antioxidant, antiviral, antibacterial, anticonvulsant, anticancer, antianxiety, antifungal, and antidepressant-like effects can lead to an understanding of its promising therapeutic effects in the medical field. However, the underlying mechanisms of these therapeutic properties are not well studied and remain undetermined. Another limitation in developing a highly effective drug is that flavonoids with a single oxygen atom in the C2′ position are extremely rare in the plant kingdom. So far, zapotin has only been established in twelve different plant species. Nevertheless, considering the fact that a deeper understanding of the effects of zapotin’s mechanisms of action, may expand the field of new therapeutics, bio-assayed isolation should be carried out.

## Figures and Tables

**Table 1:** Plants containing zapotin and their traditional uses.

Species	Family	Location	Parts of Plant	Traditional Uses	References
*Casimiroa edulis*	Rutaceae	not given	bark, seeds, leaves	hypertension, anxiety, insomnia, painkiller	[8,11,13,17,21,22,23,24,25,26,27,28,29,30,31,32]
*Casimiroa greggii*	Rutaceae	Mexico	root	not found	[33]
*Casimiroa pubescens*	Rutaceae	Mexico	seeds	antihypertensive	[34,35]
*Casimiroa tetrameria*	Rutaceae	Mexico	leaves	gastrointestinal problems	[19,20]
*Calliandra portoricensis*	Fabaceae	Nigeria	root bark	lumbago, pain, gonorrhea, anticonvulsant, antimicrobial, analgesic, antidepressant	[36,37]
*Geoffroea decorticans*	Fabaceae	Argentina	fruits	dysentery, expectorant agent, flatulence	[38,39]
*Hottonia palustris*	Primulaceae	Poland	herb	heart problems	[40,41]
*Mammea suriga*	Calophyllaceae	India	stem bark	eczema	[42,43]
*Punica granatum*	Punicaceae	Egypt	peel extract	inflammation, rheumatism, sore throat, snakebite, diabetes, burns	[44,45]
*Primula veris*	Primulaceae	Poland	leaves ^a^	respiratory, cardiac, nervous system conditions, expectorant, sedative	[46,47]
*Struthiola argentea*	Thymelaeaceae	South Africa	herb	not found	[48]
*Syzygium alternifolium*	Myrtaceae	India	fruit	gastrointestinal problems, painkiller	[49]

^a^—from cultivation and in vitro cultures.

**Table 2:** Column chromatography in the separation of zapotin.

Plant	Part of the Plant	Extract	Separation Conditions	References
*Casimiroa edulis*	seeds, trunk, and root bark	EtOH	dissolving in 4% HCl extraction with benzene CC; Al_2_O_3_; benzene:Et_2_O (4:1 *v*/*v*) crystallization from MeOH or acetone	[8,21]
		MeOH	fractionating with EtOAc CC; Silica; CHCl_3:_MeOH CC; Silica; EtOAc:petroleum ether isolation from the 25% EtOAc in Et_2_O	[58]
*Casimiroa pubescens*	seeds	EtOAc	VLC fractionation with Hex:EtOAc (7:3 *v*/*v*)	[59,60]
*Casimiroa tetrameria*	leaves	EtOH	2. extraction with EtOAc 3. CC; Sephadex LH_20_; el: MeOH 4. RPC18; MeOH, 50–100% 5. RPC18; ACN:MeOH:H_2_O	[19]
*Primula veris*	leaves	CHCl_3_	CC; cellulose; MeOH:H_2_O (7:3 *v*/*v*) CC; polyamide; MeOH prep. TLC; Silica; *n*-Hex:EtOAc (7:3 *v*/*v*) prep. TLC; Avicel; acetone:H_2_O (3:17 *v*/*v*) CC; Polyamide; MeOH CC; Sephadex LH_20_; MeOH	[45,61]
*Struthiola argentea*	herb	MeOH	fractionation with Hex and MeOH:H_2_O, CH_2_Cl_2_ and MeOH:H_2_O RPC8; ACN:H_2_O RPC18; MeOH:H_2_O	[48]

**Table 3:** High-performance liquid chromatography in the separation of zapotin.

Plant	Part of the Plant	Extract	Column	Mobile Phase	Conditions	References
*Casimiroa pubescens*	seeds	MeOH	Restek Pinnacle C18	5% AcOH and H_2_O:MeOH	30–90% B: 0–50 min, 90% B: 50–55 min	[34]
*Casimiroa portoricensis*	root bark	MeOH	not given	not given	not given	[36]
*Struthiola argentea*	herb	Hex/CH_2_Cl_2_	Zorbax RX-C8, Zorbax RX-C18	0.1% THF and H_2_O:ACN	25–100%	[48]

**Table 4:** Gas chromatography in the separation of zapotin.

Plant	Part of the Plant	Extract	Column ^a^	Conditions	References
*Casimiroa pubescens*	root	not given	not given (0.25 × 30.0 × 0.25)	30 to 310 °C; 8 °C/min; 6 min at 310 °C	[60]
*Syzygium alternifolim*	fruit	MeOH	VF-5MS (0.25 × 30.0 × 0.25)	3 min at 70 °C; 10 °C/min to 240 °C, then 5 °C/min to 300 °C, 9 min at 300 °C	[49]
*Mammea suriga*	stem bark	petroleum ether	RESTEK Rtx-5 (0.25 × 30.0 × 0.25)	3 min at 70 °C; 10 °C/min to 240 °C, then 5 °C/min to 300 °C, 9 min at 300 °C	[42]
*Punica granatum*	peel	not given	Agilent HP-5ms (0.25 × 30.0 × 0.25)	2 min at 60 °C; 10 °C/min to 280 °C	[44]

^a^—ID [mm], L [m], F [µm]).

**Table 5:** Bioactivities of zapotin reported in experimental models in vitro and in vivo.

Activity	Experimental Model	Exposure/ Incubation	Concentration	Efficacy	References
Anti-viral	RDDP5 assay	not given	70% ethanolic extracts from *C. edulis*	IC_50_ (µg/mL): HIV-1 RT RDDP: 0.27 HIV-1 RT RNase H: 2.0	[64]
Anti-cancer	K562 cell line	not given	70% ethanolic extracts from *C. edulis*	CC_50_ (µg/mL): K562 cells: 0.00031	[64]
	HL-60 cell line	not given	not given	induction of differentiation corelated with proliferation arrest ED_50_ < 8 mg/mL lack of cytotoxicity	[65]
	LNCaP, DU-145, lung adenocarcinoma, healthy VERO cell lines	24 h incubation with extracts, 72 h at 37 °C with medium, 2–4 h at 37 °C with MTS	MeOH fraction of *C. portoricensis*	Inhibition of proliferation (IC50): LNCaP: 2.4 ± 0.2 µg/mL DU-145: 3.3 ± 0.2 µg/mL lung adenocarcinoma: 3.6 ± 0.2 µg/mL healthy VERO cells: 17.9 ± 1.6 µg/mL 3-fold decreased expression of Bcl-2 and a 4-fold increase in Bax levels at 10 µg/mL in LNCaP cells 3.5-fold reduction in FIR and 4.2-fold induction of CCR at 10 µg/mL	[36]
	A549 cell line	not given	Zapotin, MeOH extract of *C. portoricensis*	inhibition of the growth of neoplast cells through an indirect pathway at the protein level	[66]
	disk diffusion assay for cytotoxicity, Colon38, L1210, MCF-7, U251N, PANC-1, H-116 cell lines	not given	1 μg/disk	potent cytotoxicity with significant solid tumor selectivity (Colon38, L1210) _MCF-7_Δ_CEM_: 250 zone units _U251N_Δ_CEM_: 400 zone units _PANC-1_Δ_CEM_: 400 zone units _H-116_ Δ_CEM_: 450 zone units	[7]
	female CD-1 mice, 4 weeks old	15 weeks	1, 5, and 10 μmol/mouse	Anti-initiation (total tumor number; average tumor number: 1 μmol: 216; 10.8 ± 6.2 5 μmol: 222; 11.1 ± 6.0 10 μmol: 138; 6.9 ± 4.9 Anti-promotion (total tumor number; average tumor number: 1 μmol: 139; 7.0 ± 5.4 5 μmol: 117; 5.9 ± 4.0 10 μmol: 123; 6.2 ± 6.3 Anti-initiation/promotion (total tumor number; average tumor number): 1 μmol: 184; 9.2 ± 4.4 5 μmol: 182; 9.1 ± 6.2 10 μmol: 163; 8.2 ± 5.7	[67]
	HeLaPKCεA/E subline	MTT assay, 72 h Western blot, 1 h 45 PKC activity	1–25 μM 7.5, 15 and 30 μM 3.75–15 μM	HeLaWT cells were treated with zapotin for 72 h and the IC_50_ value was found to be 17.9 ± 1.6 μM cytotoxic effect in cells expressing PKCεA/E activation of recombinant PKCε was dose-dependent PKCδ was down-modulated to a lesser extent by zapotin increasing doses of zapotin (3.75, 7.5, and 15 μM) attenuated the enhanced migration of doxycycline-induced cells overexpressing PKCεA/E at 15 μM, zapotin caused a significant decrease in the level of Bcl-2 by almost 40% compared to the control increasing concentration of zapotin (from 7.5 to 30 mM) caused the formation of autophagosomes and a decline in microtubule-associated protein 1 light chain 3 protein levels	[9,68]
	SW480, SW620,HT-29 cell lines, female CF-1 mice	24 h in the proliferation assay 6, 18, 24, 48 h in flow cytometry	5.0 or 10.0 mg/kg BW in the induction of ACF, 1.0 µM zapotin in flow cytometry	antiproliferative properties with HT-29 cells: IC_50_ at 212 ng/mL for the isolated zapotin compared with 192 ng/mL for the synthetic zapotin zapotin mediated growth inhibition in a dose-dependent manner, with 78% inhibition at 1 µM and an IC_50_ of 2.74 × 10^–7^ M (HT-29), 2.29 × 10^–7^ M (SW480), 5.27 × 10^–7^ M (SW620) maximum antiproliferative response of zapotin was observed after 5 days treatment with 1 µM (48 h) increased the percentage of apoptotic cells in all three cell lines reduction of ACF by 56% and 67% by zapotin at doses of 5.0 and 10.0 mg/kg zapotin treatment resulted in a reduction in the number of large ACF by 87% and 67% at doses of 5.0 and 10.0 mg/kg BW, respectively	[55]
	ODC, HepG2 and HL-60 cell lines	18 h in TPA-induced ODC, cell differentiation 48 h in TPA-induced NF-κB assay 24 h in the quantification of apoptosis and cell cycle	12 μM zapotin in the quantification of apoptosis	inhibition of the induction of ODC activity by TPA (IC_50_ = 3.4 ± 1.7 μM) inhibition of TPA-induced NF-κB activity in HepG2 cells stably transfected with NF-κB-luciferase plasmid with an IC_50_ value of 7.6 ± 3.3 μM significant increase in apoptosis at 3 μM and higher suppression in the G2/M phase of the cycle at 0.75 μM	[56]
Anti- mutagenic	Aroclor 1254-pretreated rats	not given	not given	DMBA-induced mutagenesis with *S. typhimurium* strain TM677: >40 µg/mL DMBA-induced preneoplastic lesions with MMOC: 50 µg/mL	[58]
Vasorelaxant	adhering tissue and arterial rings from 6-month-old rats	45 min before viability	*Casimiroa* spp. extracts, 20 μg/mL per se	*C. calderoniae* decreased the constriction of arterial rings by 37.5 ± 5.0% dilatation of arterial tissues: *C. edulis*: 86.1 ± 2.5% *C. calderoniae*: 95.4 ± 0.9% *C. pubescens*: 82.2 ± 6.6%	[34]
Antimicrobial	suspensions of microorganisms containing 10^6^ CFU/mL	72 h at 37 °C	water juice peel from *Punica* *granatum*	inhibition zones’ diameter (mm): *S. aureus*: 22 ± 1.1 *S. typhimurium*: 18 ± 0.2 *E. coli*: 22 ± 0.9 *A. flavus*: 11 ± 1.1 *A. parasiticus*: 15 ± 0.5 *A. niger*: 14 ± 1.0 *P. digitatum*: 17 ± 1.2	[44]
Anti- depressant-like	mice	first dose 60 min before testing, second dose 24, 7, and 1 h before testing	hexane (HCP), ethyl acetate (ECP), and methanol (MCP) extracts from *C. pubescens*	induced a reduction in the FST assay HCP at a 30 mg/kg dose; the animals showed a decrease in ambulatory activity and loss of motor coordination MCP did not produce any change in behavior in the experimental animals ECP caused a dose-dependent response in doses in the range of 7.5, 15, and 30 mg/kg; the immobility time was almost the same as that of 60, 90, and 120 mg/kg	[60]

## Data Availability

Data are contained within the article.

## Abbreviations

ACF	aberrant crypt foci
AcOH	acetic acid
ADMET	absorption, distribution, metabolism, excretion, and toxicity
Al_2_O_3_	aluminum oxide
BW	body weight
CC	column chromatography
CFU	colony-forming unit
CHCl_3_	chloroform
Et_2_O	diethyl ether
EtOAc	ethyl acetate
F	film thickness
FST	forced swim test
HCl	hydrochloric acid
Hex	hexane
HPLC	high-performance liquid chromatography
HPLC-MS	high-performance liquid chromatography–mass spectrometry
HPMFs	hydroxypolymethoxyflavones
ID	inner diameter
IR	infrared radiation
L	length
LC-MS	liquid chromatography–mass spectrometry
LC-MS-MS	liquid chromatography–tandem mass spectrometry
LiHMDS	lithium hexamethyldisilazide
MeOH	methanol
MMOC	mouse mammary organ culture
mp	melting point
NMR	nuclear magnetic resonance spectroscopy
ODC	ornithine decarboxylase
PMFs	polymethoxyflavones
PKC	protein kinase C
Rf	retention factor
RP	reversed phase
SFC	supercritical fluid chromatography
t_0.5_	time of half-life
THF	tetrahydrofurane
TPA	12-*O*-tetradecanoylphorbol-13-acetate
UV	ultraviolet
VLC	vacuum liquid chromatography
